# Supplementary material for: Quantifying Induced Dipole Effects in Small Molecule Permeation in a Model Phospholipid Bilayer
Source: J Phys Chem B. 2024 Jul 22;128(30):7385–400. doi: 10.1021/acs.jpcb.4c01634 (PMC11301690; doi:10.1021/acs.jpcb.4c01634)
Supplement: Supplementary file 1 — jp4c01634_si_001.pdf [file jp4c01634_si_001.pdf]

SUPPORTING INFORMATION

Quantifying Induced Dipole Effects in Small Molecule Permeation in a Model Phospholipid Bilayer

Julia M. Montgomery[1] and Justin A. Lemkul[1,2]

[1]Department of Biochemistry, [2]Center for Drug Discovery
Virginia Tech, Blacksburg VA 24061, United States

SUPPORTING TABLES

Table S1. Simulation times (ns) for umbrella sampling windows in each system.

	**CHARMM36**	**Drude**
Isobutane	50	70
Benzene	80	50
Indole	50	90
Methylimidazole	50	50
Ethanethiol	50	50
Ethanol	50	80
Acetate	50	70
Methylguanidinium	50	50

Table S2. Average molecular dipole moments (D) of solute molecules in bulk water.

	**CHARMM36**	**Drude**
Isobutane	0.145	0.574
Benzene	0.173	0.634
Indole	2.259	3.461
Methylimidazole	4.612	5.871
Ethanethiol	1.450	2.312
Ethanol	2.413	2.781
Acetate	4.342	6.164
Methylguanidinium	1.807	2.023

SUPPORTING FIGURES

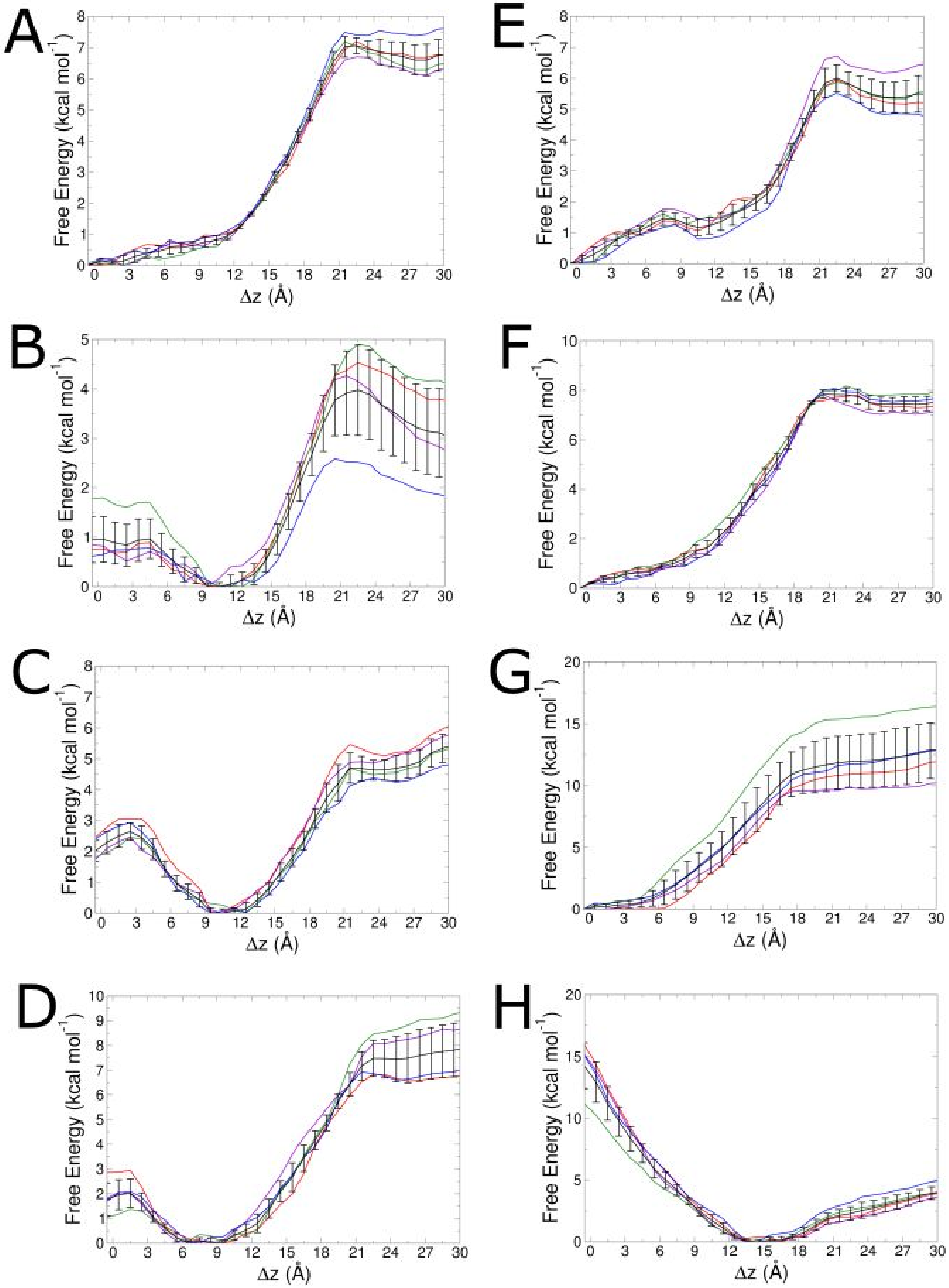

Figure S1. Convergence checks for C36 simulations. Free energy surfaces from last 40 ns in 10 ns intervals are plotted in different colors (in order: green, blue, purple, red) along with the pooled data (black with error bars) for (A) isobutane, (B) benzene, (C) indole, (D) methylimidazole, (E) ethanethiol, (F) ethanol, (G) acetate, (F) methylguanidinum.

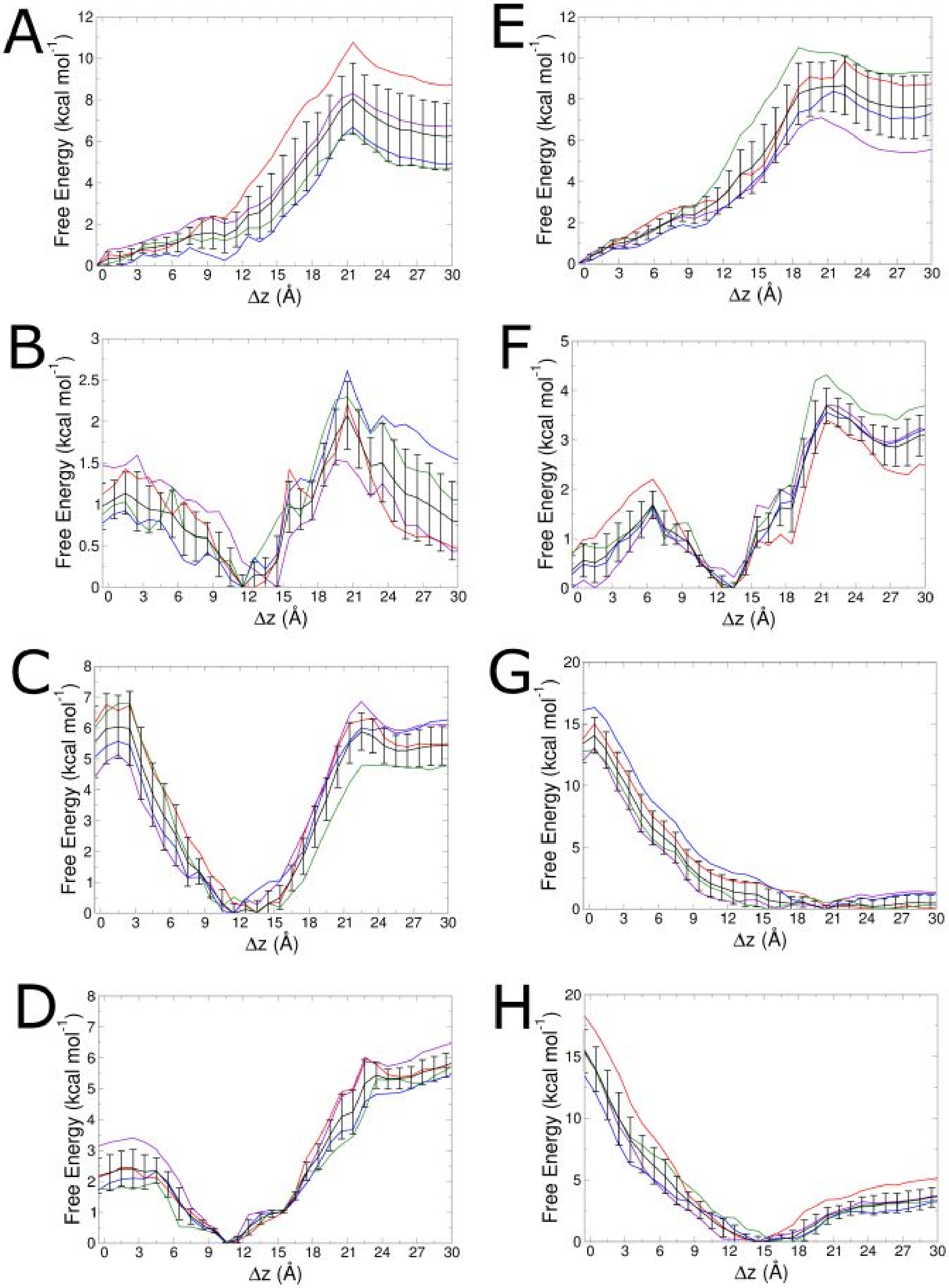

Figure S2. Convergence checks for Drude simulations. Free energy surfaces from last 40 ns in 10 ns intervals are plotted in different colors (in order: green, blue, purple, red) along with the pooled data (black with error bars) for (A) isobutane, (B) benzene, (C) indole, (D) methylimidazole, (E) ethanethiol, (F) ethanol, (G) acetate, (F) methylguanidinum.

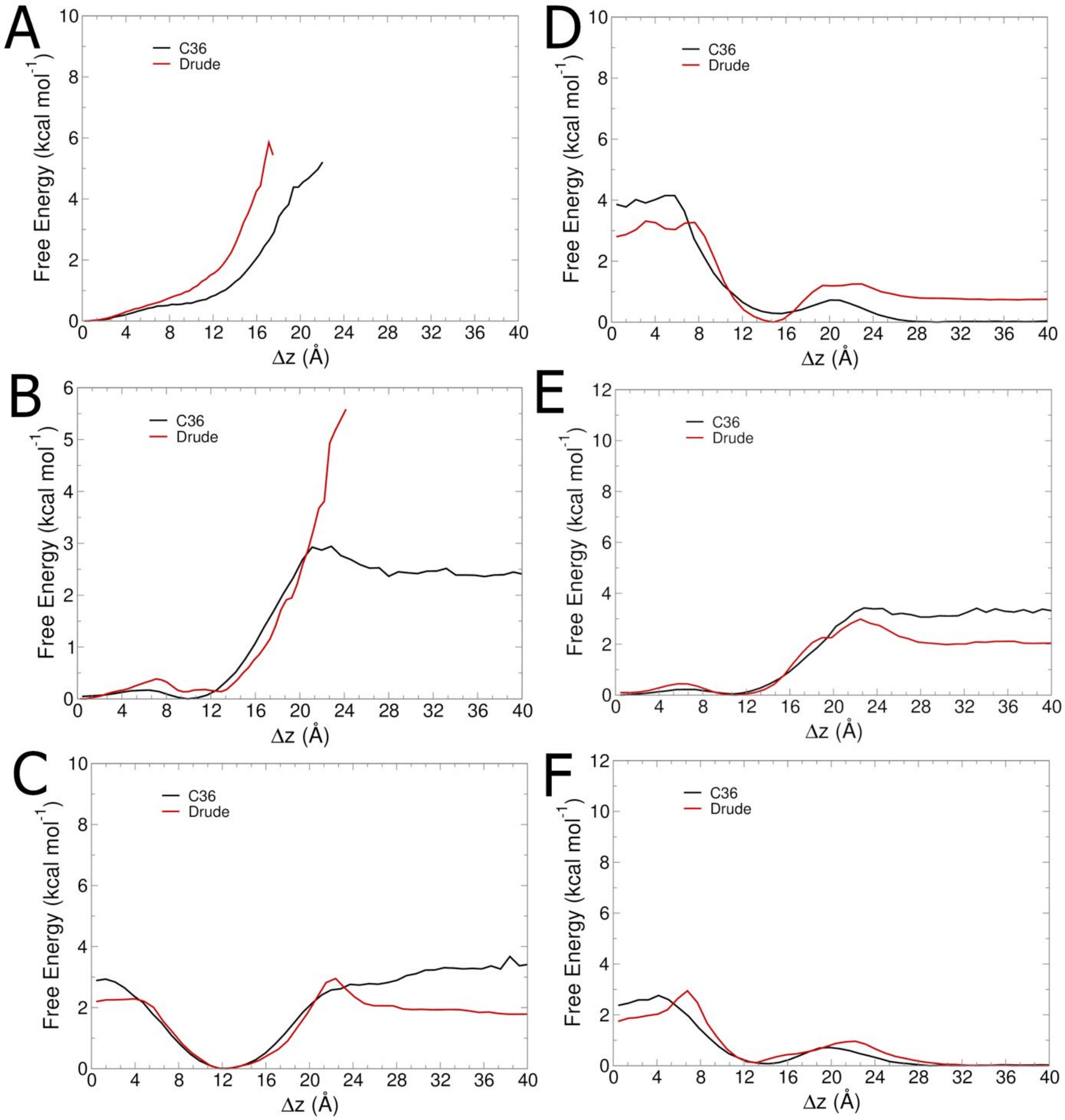

Figure S3. Free energy surfaces derived from unbiased simulations for (A) isobutane, (B) benzene, (C) indole, (D) methylimidazole, (E) ethanethiol, (F) ethanol.

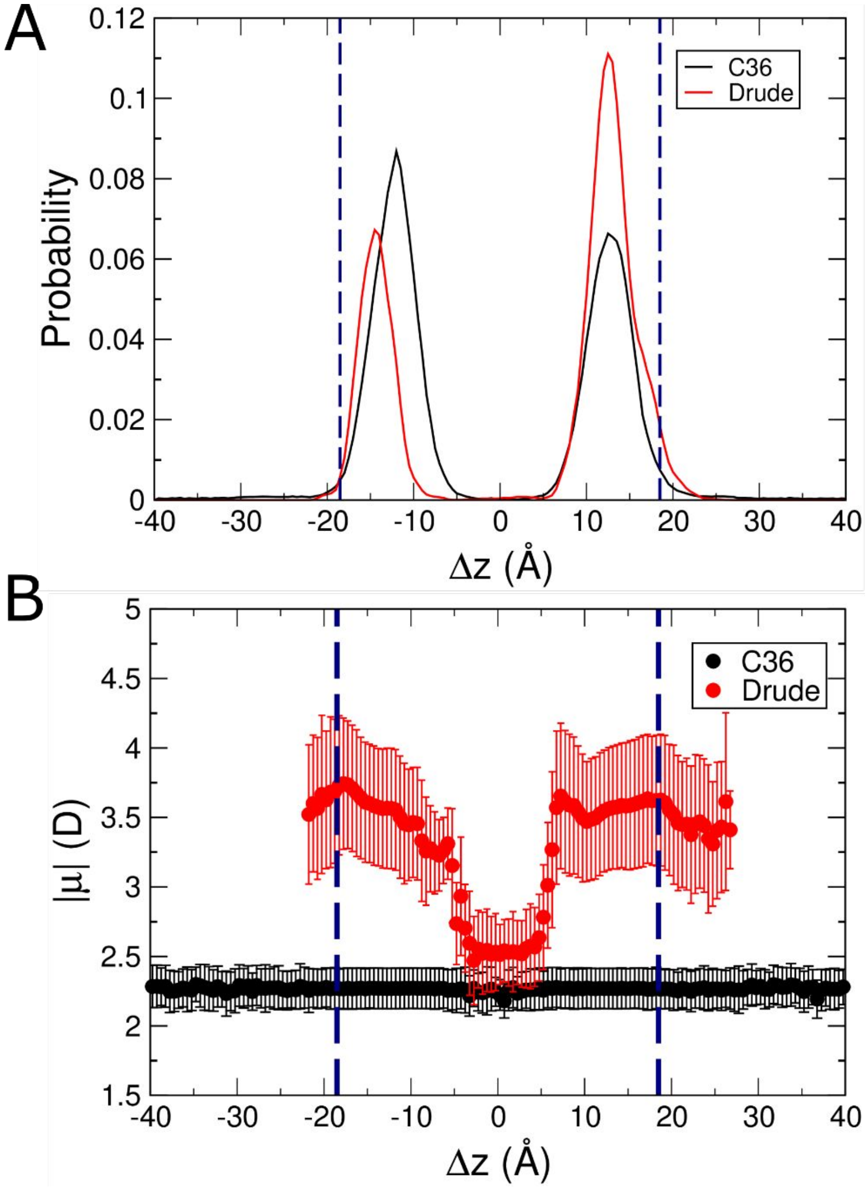

Figure S4. Results from unbiased simulations of indole starting from the flipped orientation. (A) Normalized probability of localization of indole in unbiased simulations. (B) Molecular dipole moments as a function of position within the membrane in unbiased simulations. Error bars correspond to the root-mean-squared fluctuation (RMSF) of the binned data points.

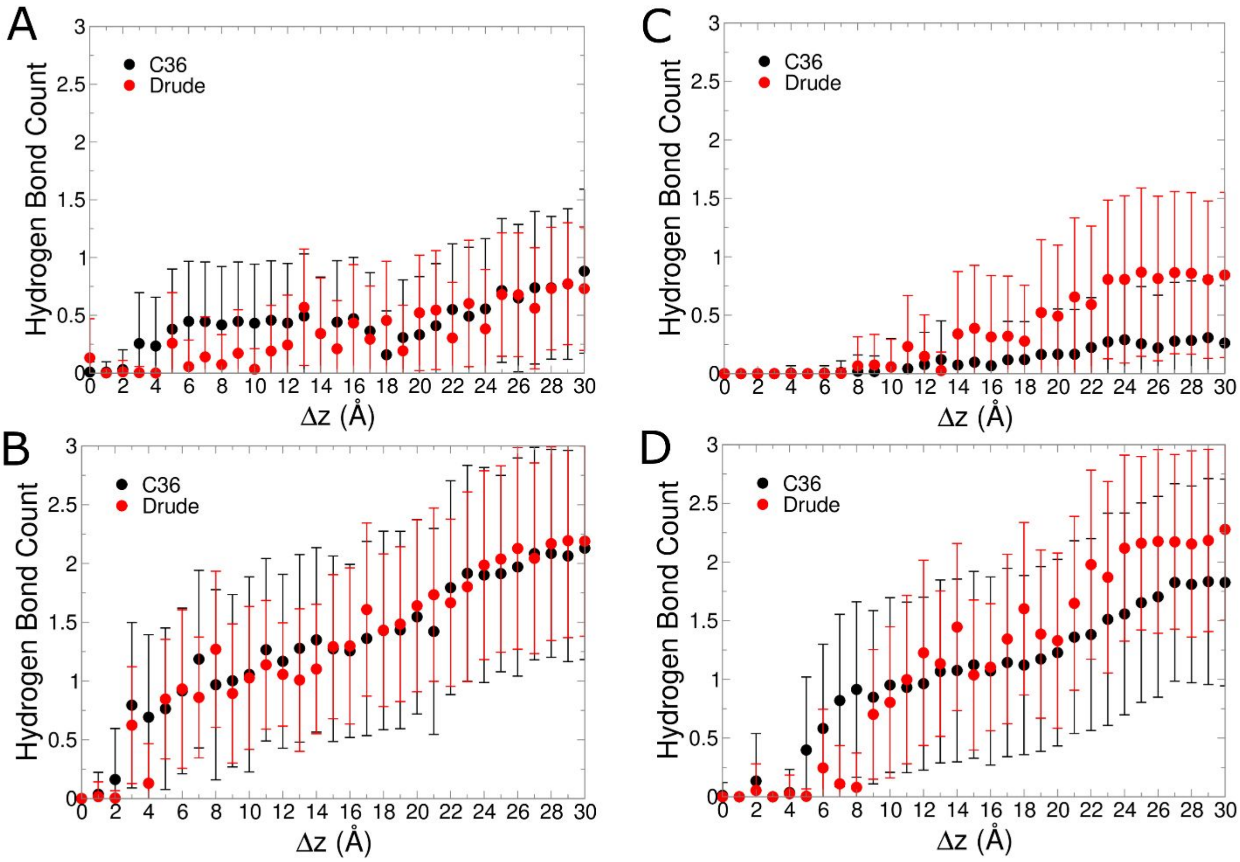

Figure S5. The average number of hydrogen bonds occurring between water and the small molecule in each umbrella sampling window. (A) Indole, (B) methylimidazole, (C) ethanethiol, and (D) ethanol. Error bars correspond to the root-mean-squared fluctuation (RMSF) of the binned data points.

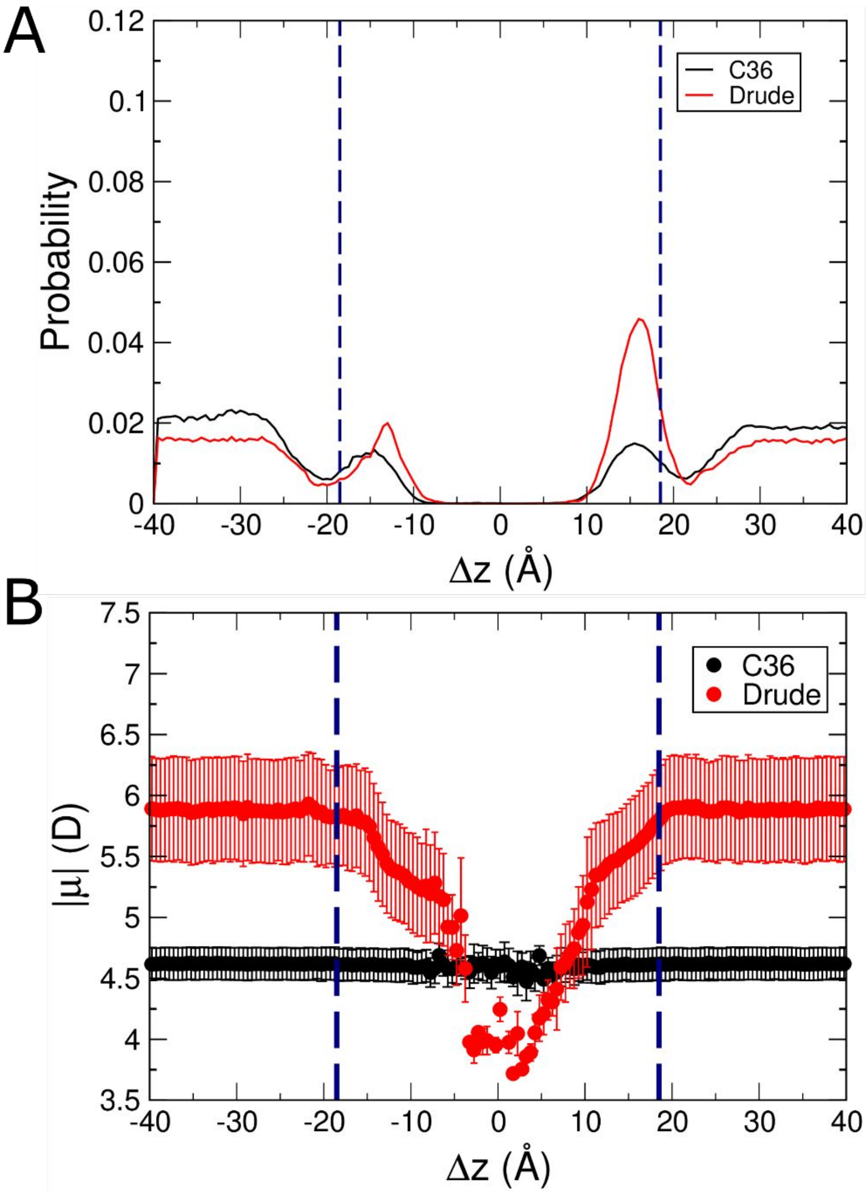

Figure S6. Results from alternate unbiased simulations of methylimidazole starting from the flipped orientation. (A) Normalized probability of localization of methylimidazole in unbiased simulations. (B) Molecular dipole moments as a function of position within the membrane in unbiased simulations. Error bars correspond to the RMSF of the binned data points.

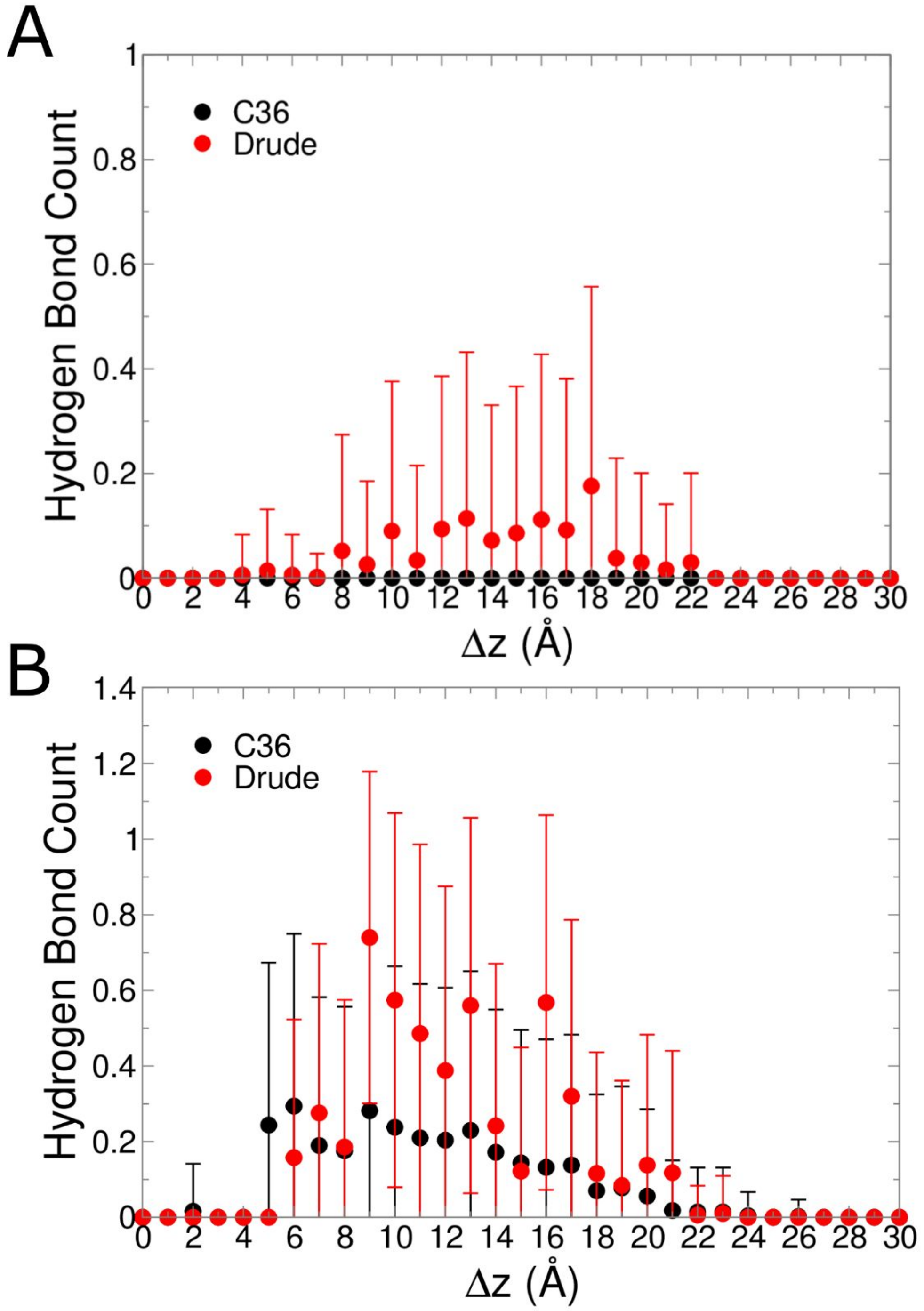

Figure S7. The average number of hydrogen bonds formed between POPC and the small molecule in each umbrella sampling window. (A) Ethanethiol, and (B) ethanol. Error bars correspond to the root-mean-squared fluctuation (RMSF) of the binned data points.

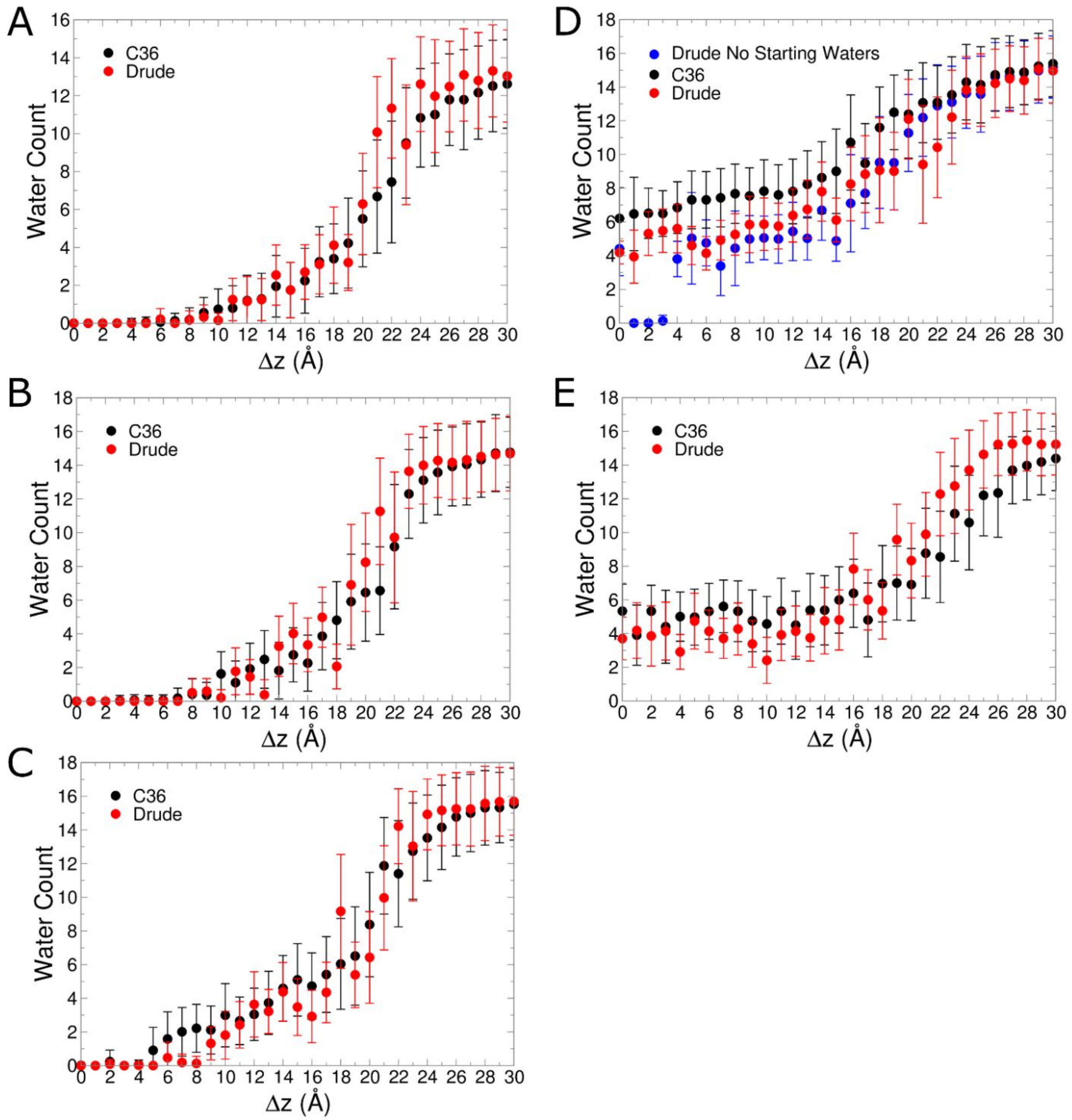

Figure S8. The average number of water molecules within 5 Å of the center of mass of each small molecule in each umbrella sampling window. (A) Benzene, (B) ethanethiol, (C) ethanol, (D) acetate (including results from the alternate starting coordinates), and (E) methylguanidinum. Error bars correspond to the root-mean-squared fluctuation (RMSF) of the binned data points.
